# Calycosin Suppresses RANKL-Mediated Osteoclastogenesis through Inhibition of MAPKs and NF-κB

**DOI:** 10.3390/ijms161226179

**Published:** 2015-12-10

**Authors:** Gui-Hua Quan, Hongbing Wang, Jinjin Cao, Yuxin Zhang, Donglin Wu, Qisheng Peng, Ning Liu, Wan-Chun Sun

**Affiliations:** 1 Key Laboratory for Molecular and Chemical Genetics of Critical Human Diseases of Jilin Province, Jilin University Bethune Second Hospital, Changchun 130041, China; qgh0521@jlu.edu.cn; 2 School of Life Sciences and Technology, Tongji University, Shanghai 200092, China; hbwang@tongji.edu.cn; 3 Key laboratory of Zoonosis, Ministry of Education, Institute of Zoonosis, Jilin University, Changchun 130062, China; jinjincao90@163.com (J.C.); chongfengxing@163.com (Y.Z.); pengqs@jlu.edu.cn (Q.P.); 4 Department of Virus Disease Prevention and Control, Jilin Provincial Center for Disease Control and Prevention, Changchun 130062, China; dl_wu@163.com

**Keywords:** calycosin, osteoclast, bone resorption, NFATc1, c-Fos, NF-κB, MAPKs

## Abstract

Calycosin, an isoflavonoid phytoestrogen, isolated from *Radix Astragali*, was reported to possess anti-tumor, anti-inflammation, and osteogenic properties, but its impact on osteoclast differentiation remains unclear. In this study, we examined the effects of calycosin on osteoclastogenesis induced by RANKL. The results showed that calycosin significantly inhibited RANKL-induced osteoclast formation from primary bone marrow macrophages (BMMs). Calycosin also dose-dependently suppressed the formation of bone resorption pits by mature osteoclasts. In addition, the expression of osteoclatogenesis-related genes, including cathepsin K (CtsK), tartrate-resistant acid phosphatase (TRAP), and MMP-9, was significantly inhibited by calycosin. Furthermore, the results indicated that calycosin down-regulated the expression levels of NFATc1 and c-Fos through suppressing the activation of NF-κB and MAPKs. Our results indicate that calycosin has an inhibitory role in the bone loss by preventing osteoclast formation, as well as its bone resorptive activity. Therefore, calycosin may be useful as a therapeutic reagent for bone loss-associated diseases.

## 1. Introduction

Bone remodeling is the continuous process, which is delicately controlled by the balance between bone formation by osteoblasts and bone resorption by osteoclasts [1]. Osteoclasts are bone-resorbing multinucleated cells formed by the fusion of mononuclear progenitors of the monocyte/macrophage hematopoietic lineage cells [2]. Increased numbers of osteoclasts lead to bone loss-associated disorders including rheumatoid arthritis, osteoporosis, Paget’s disease, osteosarcoma, periodontal disease, and cancer bone osteolytic metastasis [2,3,4,5].

The differentiation of osteoclasts requires the presence of both crucial cytokines, macrophage colony stimulation factor (M-CSF), and receptor activator of NF-κB ligand (RANKL) [3,6]. RANKL binding to its receptor RANK on the surface of osteoclast precursor cells leads to the recruitment of TNF receptor-associated factor 6 (TRAF6) and the subsequent activation of mitogen-activated protein kinases (MAPKs), including ERK, p38, and JNK, as well as transcription factors, such as nuclear factor κB (NF-κB), nuclear factor of activated T-cells, cytoplasmic 1 (NFATc1), and c-Fos [6,7,8,9]. The activation of above signaling pathways directly regulates the expression of osteoclastic genes, such as Cts K, MMP-9, and TRAP, which regulate the formation of bone resorption pits during osteoclast differentiation [10]. Therefore, suppressing RANKL-induced osteoclast differentiation-related signaling pathways is a potential therapeutic intervention for preventing osteoclastogenesis.

**Figure 1:**
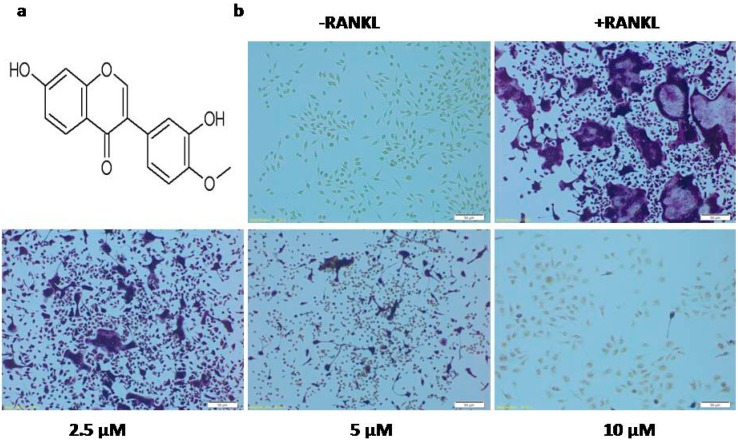

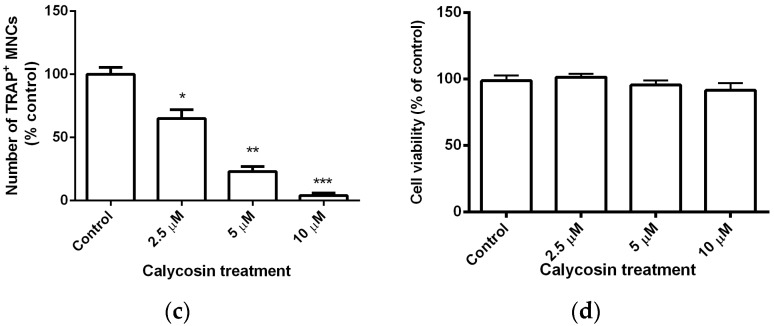
Effects of calycosin on osteoclast differentiation. (**a**) Chemical structure of calycosin; (**b**) BMMs were cultured with indicated dose of calycosin in the presence of M-CSF (20 ng/mL) and RANKL (20 ng/mL). After three days, cells were fixed and stained for TRAP staining assay; (**c**) TRAP-positiveMNCs containing three or more nuclei were counted as osteoclasts; and (**d**) cell viability was measured using MTT assay. The results were expressed as means ± SD from three independent experiments. Scale bar represents 50 μM. * *p* < 0.05; ** *p* < 0.01; *** *p* < 0.001 *vs.* control.

Calycosin (Figure 1a), the major isoflavonoid in *Radix Astragali Mongolici*, was reported to exhibit anti-tumor and anti-inflammation effects as estrogen receptor modulators (SERMs) via inhibiting NF-κB activation and MAPKs phosphorylation [11,12,13,14,15]. Calycosin also was demonstrated to be essential to osteogenic abilities of DangguiBuxue Tang, a chinese herbal decotion containg *Astragali Radix* and *Angelicae Sinensis Radix* [16], but its impact on osteoclast differentiation remains unclear. In this study, we investigated the effects of calycosin on RANKL-induced osteoclast formation and bone resorptive activity, and further clarified the underlying mechanism by which calycosin suppressed RANKL-induced osteoclast formation.

## 2. Results

### 2.1. Calycosin Inhibits RANKL-Induced Osteoclast Formation

To confirm whether calycosin can influence osteoclast differentiation, BMMs were cultured with the indicated concentration of calycosin (2.5, 5 and 10 μM) in the presence of RANKL (20 ng/mL) and M-CSF (20 ng/mL) for three days. As shown in Figure 1b, the control group formed numerous TRAP-positive multinucleated osteoclasts. In contrast, treatment with calycosin dose-dependently reduced osteoclast formation from RANKL-stimulated BMMs (Figure 1b,c). The formation of osteoclasts was almost completely diminished by calycosin at the concentration of 10 μM. These results demonstrated that calycosin inhibited RANKL-induced osteoclast formation from mouse BMMs in a concentration-dependent manner.

To exclude the possibility that calycosin inhibits osteoclast differentiation through its toxicity, the cell viability of BMMs in the presence of RANKL, alone or together with various concentrations of calycosin, was tested using MTT assay. As shown in Figure 1d, calycosin did not show significant cytotoxicity at the concentrations up to 10 μM. These results suggested that the inhibitory effects of calycosin on RANKL-induced osteoclastogenesis were not due to potential cytotoxic effects of the compound.

### 2.2. Calycosin Reduces RANKL-Induced Bone Resorption by Osteoclasts

**Figure 2:**
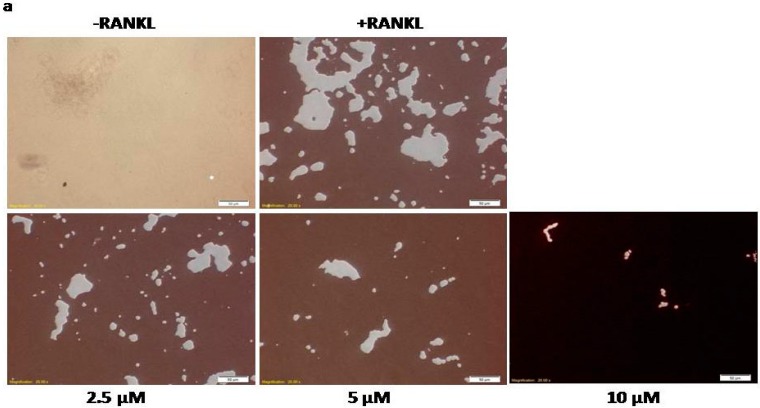

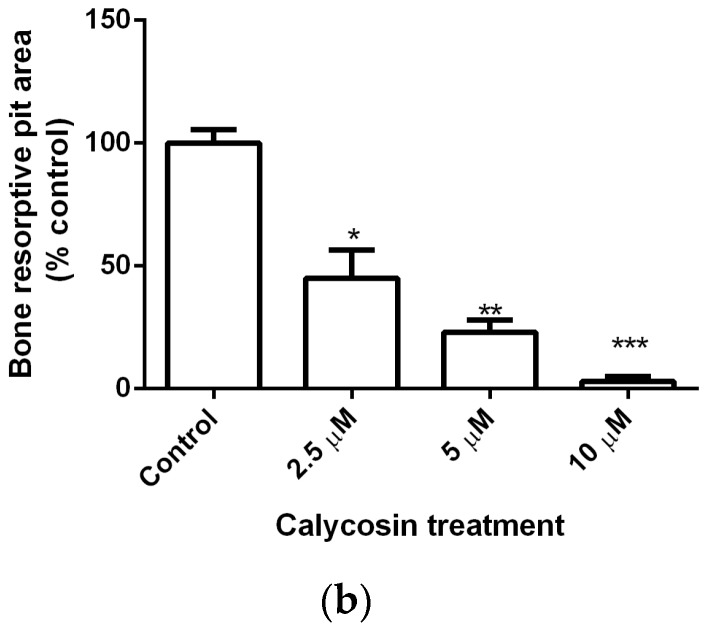
Effects of calycosin on the generation of bone resorption pits. (**a**) BMMs were cultured with indicated dose of calycosin in the presence of M-CSF (20 ng/mL) and RANKL (20 ng/mL) on Corning Osteo Assay Surface 24 well plates. After six days, cells were removed using 5% sodium hypochlorite and staining by Von Kossa method with modified; and (**b**) the areas were quantified by Image J. The results were expressed as means ± SD from three independent experiments. Scale bar represents 50 μM. * *p* < 0.05; ** *p* < 0.01; *** *p* < 0.001 *vs.* control.

To further confirm the inhibitory effects of calycosin on RANKL-induced osteoclastogenesis, we examined the effects of calycosin on bone resorption by culturing RANKL-stimulated BMMs on a calcium phosphate-coated Corning Osteo Assay Surface *in vitro* model system. The results showed calycosin dose-dependently reduced bone resorption pit formation by RANKL-stimulated BMMs, and at a concentration of 10 μM, calycosin almost completely prevented resorption pit formation by RANKL-stimulated BMMs (Figure 2). Collectively, these findings demonstrated that calycosin attenuated bone resorption by osteoclasts *in vitro* in a concentration-dependent manner.

### 2.3. Calycosin Inhibits RANKL-Induced Expression of Osteoclastic Marker Genes

**Figure 3:**
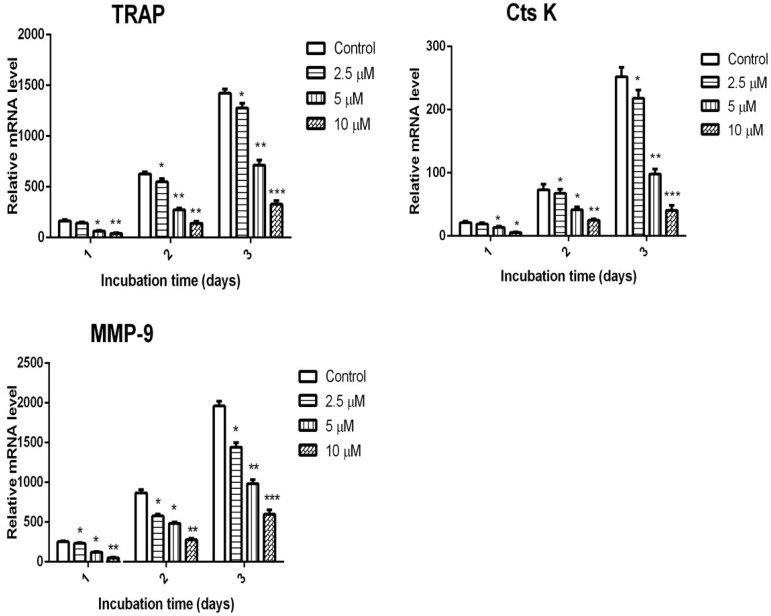
Effects of calycosin on RANKL-induced gene expression. BMMs were cultured with 20 ng/mL of M-CSF and 20 ng/mL of RANKL in the presence or absence of indicated dose of calycosin for one, two, or threedays. The mRNA expression levels of the indicated genes including TRAP, CtsK, and MMP-9 were determined by quantitative real-time PCR. The mRNA levels of the target gene were normalized relative to the mRNA level of β-actin. The results were expressed as means ± SD from three independent experiments. * *p* < 0.05; ** *p* < 0.01; *** *p* < 0.001 *vs.* control.

We further examined the expression profiles of osteoclastic genes such as TRAP, MMP-9, and Cts K. As shown in Figure 3, the mRNA expression levels of TRAP, MMP-9, and Cts K were significantly increased by RANKL stimulation. However, the induction of these genes was dramatically inhibited by the presence of calycosin. We further confirmed that calycosin dose-dependently suppressed RANKL-induced expression of osteoclastic mark genes. Collectively, these results suggested that calycosin suppressed RANKL-induced osteoclast differentiation in a dose-dependent manner.

### 2.4. Calycosin Suppresses RANKL-Induced c-Fos and NFATc1 Expression

**Figure 4:**
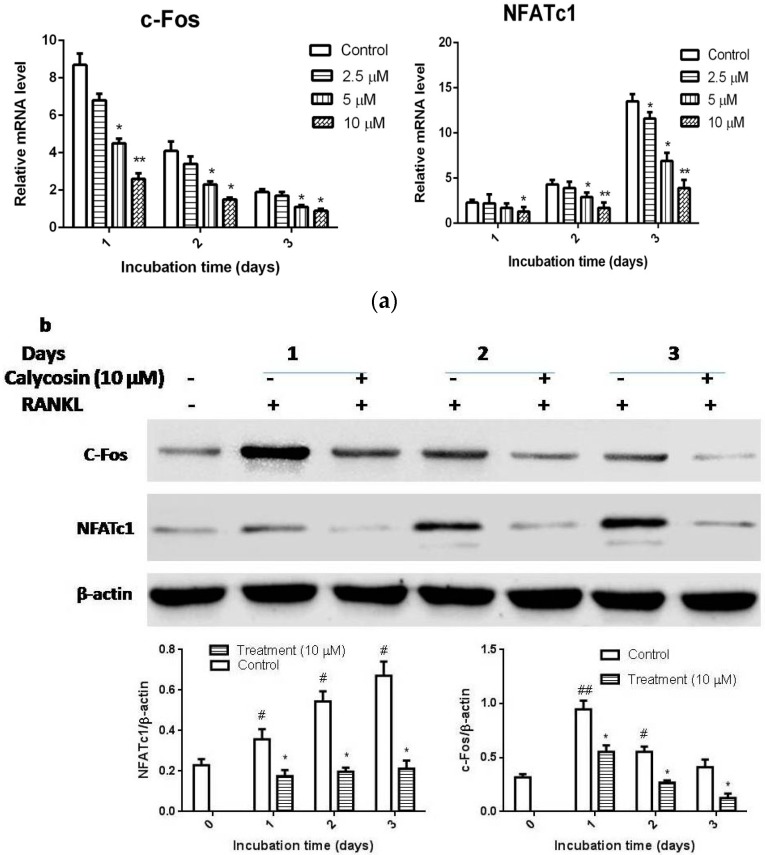
Effects of calycosin on the expression of c-Fos and NFATc1. BMMs were cultured with 20 ng/mL of M-CSF and 20 ng/mL of RANKL in the presence or absence of indicated dose of calycosin for one, two, or three days. (**a**) The mRNA expression levels of c-Fos and NFATc1 were examined by quantitative real-time PCR; and (**b**) the protein levels of c-Fos and NFATc1 were examined by western blot. The relative quantification of target proteins was calculated by comparison of the bands density levels between samples. The results were expressed as means ± SD from three independent experiments. * *p* < 0.05 *vs.* control. ^#^ *p* < 0.05 and ^##^ *p* < 0.01 *vs.* vehicle-treated group.

To determine the underlying molecular mechanism, we further evaluated the effects of calycosin on the expression levels of crucial transcription factors, such as NFATc1 and c-Fos. As reported previously, the expression levels of c-Fos and NFATc1 were increased in RANKL-stimulated BMMs. As shown in Figure 4a, calycosin apparently inhibited RANKL-induced mRNA expression of c-Fos and NFATc1 and also reduced the protein expression levels of both transcription factors (Figure 4b). These results suggested that calycosin suppressed RANKL-mediated osteoclastogenesis via inhibiting the expression of key transcription factors such as c-Fos and NFATc1 in a dose-dependent manner.

### 2.5. Calycosin Inhibits RANKL-Induced NF-κB Activation

**Figure 5:**
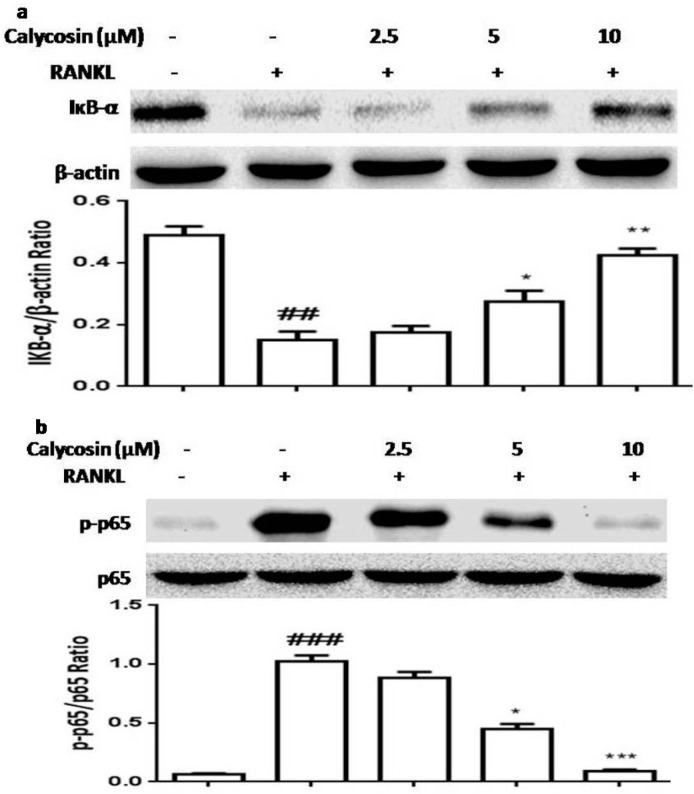
Effects of calycosin on the activation of NF-κB by RANKL. BMMs were pretreated with the indicated dose of calycosin or DMSO (vehicle) for 20 min, then RANKL (20 ng/mL) was added for 30 min. (**a**) The effects of calycosin on the degradation of IкB-α; and (**b**) the effects of calycosin on the phosphorylation levels of NF-κB p65 subunit. The relative quantification of target proteins was calculated by comparison of the bands density levels between samples. The values shown in the graphs were the mean ± SD (*n* = 3). ^##^ *p* < 0.01 and ^###^ *p* < 0.001 *vs.* vehicle-treated group; * *p* < 0.05; ** *p* < 0.01; *** *p* < 0.001 *vs.* RANKL-treated group.

NF-κB is an important transcription factor promoting an initial induction of NFATc1 then causing osteoclastogenesis and the transcription of genes encoding osteoclastogenesis-related inflammatory mediators [2,3]. NF-κB is one of the downstream transcription factors of RANKL in osteoclasts and it regulates the transcription of a number of target genes. To verify whether calycosin suppressesRANKL-induced expression of c-Fos and NFATc1 by blocking NF-κB signaling in BMMs, we examined the effects of calycosin on RANKL-induced NF-κB activation. As shown in Figure 5, calycosin dose-dependently inhibited RANKL-induced IκB-α degradation and NF-κB p65 subunit phosphorylation. These results demonstrated calycosin inhibited RANKL-mediated osteoclastogenesis via blocking NF-κB activation.

### 2.6. Calycosin Inhibits RANKL-Induced MAPK Phosphorylation

**Figure 6:**
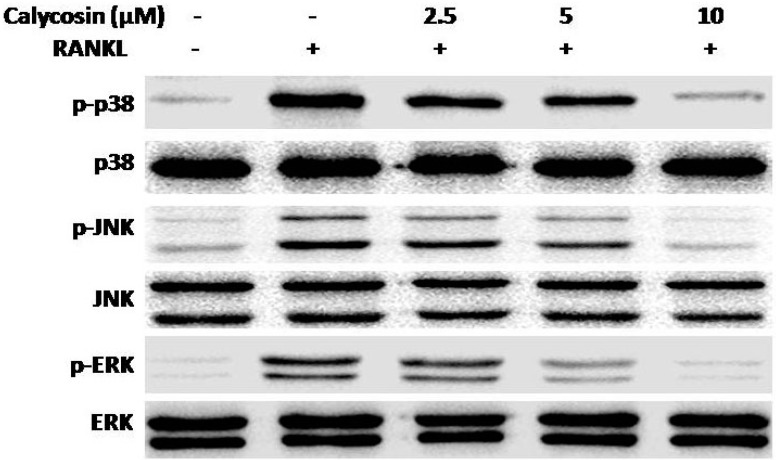

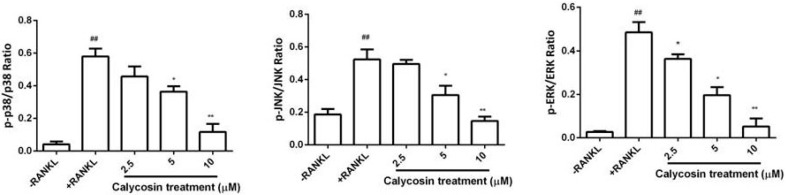
Effects of calycosin on the phosphorylation of MAPKs by RANKL. BMMs were pretreated with the indicated dose of calycosin or DMSO (vehicle) for 20 min, then RANKL (20 ng/mL) was added for 30 min. The relative quantification of target proteins was calculated by comparison of the bands density levels between samples. The values shown in the graphs were the mean ± SD (*n* = 3). ^##^ *p* < 0.001 *vs.* vehicle-treated group; * *p* < 0.05 and ** *p* < 0.01 *vs*. RANKL-treated group.

Activation of the MAPKs pathway plays a pivotal role in osteoclastogenesis [17,18,19,20]. To further elucidate the molecular mechanism underlying the inhibitory effects of calycosin on RANKL-induced osteoclastogenesis, we examined whether calycosin affects RANKL-induced MAPKs activation. As shown in Figure 6, calycosin significantly suppressed the phosphorylation of ERK1/2, p38 and JNK in a dose-dependent manner, but did not alter the expression of total ERK1/2, p38 and JNK. These results demonstrated calycosin also inhibited RANKL-mediated MAPKs activation in osteoclasts.

## 3. Discussion

Previous studies have found that some phytoestrogens, such as genistein, formononetin, 8-Prenylnaringenin, daidzein, and resveratrol were reported to possess anti-osteoporotic potential through suppressing osteoclastogenesis [21,22,23,24,25]. Calycosin, an isoflavonoid phytoestrogen isolated from *Radix Astragali Mongolici*, was reported to have osteogenic abilities [16], but its effects on osteoclastogenesis remains unclear. The present study demonstrated, for the first time, that calycosin was capable of inhibiting osteoclast differentiation and bone resorption via suppression of RANKL-induced NF-κB and MAPKs signaling pathways.

Several proteolytic enzymes, including TRAP, Cts K, and MMP-9, have been demonstrated to play important roles in degrading the organic bone matrix. Both Cts K and MMP-9, which are secreted by osteoclasts, are proteases to degrade collagens in hard tissue during osteoclastic resorption [10]. RANKL-stimulated osteoclast precursor cells undergo bone resorption because of the interaction of proteins produced by matured osteoclasts (TRAP, MMP-9, and Cts K). Herein, RANKL stimulation in BMMs led to the formation of many resorption pits on a Corning Osteo Assay Surface and calycosin treatment significantly reduced the formation of resorption pits. Results from real-time PCR further revealed that the anti-bone resorption effects of calycosin were accompanied by decreased expression levels of TRAP, Cts K and MMP-9.

The binding of RANKL to RANK leads to robust induction of c-Fos and NFATc1, which are key transcription factors for RANKL-induced osteoclast differentiation. The crucial role of the c-Fos in osteoclastogenesis has been revealed by knock-out experiments. The c-Fos deficient mice exhibit a severe osteopetrosis due to the failure of osteoclast differentiation [26,27]. NFATc1, a master regulator of osteoclast differentiation, regulates a number of osteoclast specific genes, such as TRAP, Cts K, and MMP-9, through cooperation with MITF and c-Fos [28]. In addition, previous study demonstrated that NFATc1-deficient embryonic stem cells are unable to differentiate into osteoclasts in response to RANKL, and the forced expression of NFATc1 leads to the formation of osteoclasts from BMMs in the absence of RANKL [9]. In this study, our results indicated that calycosin significantly suppressed the RANKL-induced c-Fos and NFATc1 expression at mRNA and protein level. These results suggested that calycosin inhibited RANKL-induced expression of osteoclastic mark genes, including TRAP, Cts K, and MMP-9, through down-regulating the expression of both c-Fos and NFATc1.

Studies have revealed that MAPKs, including JNK, ERK1/2, and p38, play a crucial role in osteoclastogenesis. It has been confirmed that binding of RANKL to RANK activates MAPK signaling, ultimately promotes osteoclastogenesis-related gene expression [3,29]. Previous reports demonstrated that specific inhibition of p38, JNK, and ERK1/2 attenuated RANKL-induced osteoclastogenesis [17,30,31,32]. Our results showed that calycosin dose-dependently suppressed the phosphorylation of ERK1/2, p38, and JNK, and calycosin exhibited stronger inhibitory effect on ERK1/2 phosphorylation. These results suggested that calycosin may down-regulate RANKL-induced c-Fos expression via blocking MPAKs phosphorylation.

NF-κB signaling also plays an indispensable role in osteoclastogenesis by promoting an initial induction of NFATc1 and the transcription of genes encoding osteoclastogenesis-related inflammatory mediators. Previous study demonstrated that selective inhibiting NF-κB activation blocked osteoclastogenesis and prevented inflammatory bone destruction both *in vivo* and *in vitro* [33]. In the present study, we found that pretreatment with calycosin suppressed IκB-α degradation and NF-κB p65 subunit phosphorylation. The results suggested that calycosin may down-regulate NFATc1 expression and inhibit RANKL-mediated osteoclastogenesis via preventing NF-κB activation.

## 4. Experimental Section

### 4.1. Reagents

Calycosin (purity > 99%) was isolated from the root of *Radix Astragali Mongolici* by the School of Life Sciences and Technology, Tongji University, and the NMR Spectrum and MS Data for Calycosin were shown in Figure S1–S3. Penicillin/streptomycin, fetal bovin serum (FBS), α-minium essential medium (α-MEM) and phosphate-buffered saline (PBS) were purchased from Thermo Scientific (Beijing, China). Phosphatase inhibitor cocktail, protease inhibitor cocktail, the leukocyte acid phosphatase kit 386A (TRAP staining kit), and 3-(4,5-dimethylthiazol-2-yl)-2,5-diphenylterazolium (MTT) were purchased from Sigma (St. Louis, MO, USA). Cell lysis buffer and antibodies against phospho-p65, p65, phospho-p38, p38, phospho-ERK1/2, ERK1/2, phospho-JNK, JNK, β-actin, IкB-α, NFATc1, and c-Fos were purchased from Cell Signaling Technology (Beverly, MA, USA). Recombinant mouse M-CSF was purchased from Peprotech (Rocky Hill, NJ, USA). Total RNA Mini-Pres Kit was obtained from Sangong Biotech (Shanghai, China). Recombinant mouse soluble RANK ligand (RANKL) was from Prospec (Rehovot, Israel). Faststart Universal SYBR Green Master (ROX) and First Strand cDNA Synthesis Kitwere purchased from Roche Diagnostics (Indianapolis, IN, USA). All other chemicals were commercial products of reagent grade.

### 4.2. Cell Culture

Bone marrow cells were obtained from male C57BL/6 mice of six weeks old, which were sacrificed using ether anesthesia as previously described [34]. Briefly, murine bone marrow cells collected from the tibia and femur of mice were cultured overnight in α-MEM containing 10% FBS. Non-adherent cells were collected and further cultured in α-MEM containing 10% FBS and 20 ng/mL M-CSF for three days. Cells that adhered to the bottom of the dish were classified as bone marrow derived macrophages (BMMs). All animal experiments were approved by “Institutional Animal Ethics Committee”, of Jilin University and performed in accordance with the Jilin university ethnic committee guideline for the Care and Use of Laboratory Animal (SCXK 2015-0004, 2015-01-07).

### 4.3. Assessment of Cell Viability

BMMs were seeded in a 96-well plate at a density of 4 × 10^4^ cells/well and incubated in α-MEM with 10% FBS, 20 ng/mL M-CSF, 20 ng/mL RANKL and the indicated concentration of calycosin for 24 h. MTT solution was added to each well followed by incubation for 4 h. After removing the medium, DMSO was added to dissolve the formazan dye. The optical density (OD) at 570 nm was measured with a microplate reader (Tecan, San Jose, CA, USA).

### 4.4. Osteoclast Differentiation and TRAP Staining Assay

BMMs were seeded in a 48 well plate at a density of 6 × 10^4^ cells/well and incubated in α-MEM with 10% FBS, 20 ng/mL M-CSF, 20 ng/mL RANKL, and the indicated concentration of calycosin (10, 5, and 2.5 μM) for 3 days. To confirm the formation of osteoclasts, staining was performed using TRAP staining kit according to the manufacturer’s instruction. TRAP-positive cells appeared dark red, and those containing three or more nuclei were classified as osteoclasts.

### 4.5. Bone Resorption Assay and Modified Von Kossa Staining

BMMs were seeded in a Corning Osteo Assay Surface 24 well plate at a density of 1 × 10^5^ cells/well and incubated in α-MEM with 10% FBS, 20 ng/mL M-CSF, 20 ng/mL RANKL and indicated concentration of calycosin (10, 5, and 2.5 μM) for 6 days. Medium and treatment were changed every 72 h until the end of 6 days culture period. Cells were removed by incubating with 5% sodium hypochlorite for 5 min. Then the wells were stained by modified Von Kossa method as previously described [34].

### 4.6. RNA Extraction and Real-Time Quantitative RT-PCR

Osteoclasts derived from BMMs were collected during the three days of differentiation, and total RNA was isolated with Total RNA Mini-Prep Kit according to the manufacturer’s instruction. RNA (100 ng) was reverse transcribed using a First Strand cDNA Synthesis Kit according to the manufacturer’s protocol. Real-time PCR assays were carried out with Faststart Universal SYBR Green Master (ROX) in an Applied Biosystems 7500 Fast Real-Time PCR System (Foster City, CA, USA). PCR primers for real-time PCR are listed in Table 1. All primers were synthesized from Sangong Biotech (Shanghai, China). Relative levels of tested genes were normalized to that of β-actin. In conventional RT-PCR, all primers generated only one amplification band resolved by agarose gel electrophoresis and were visualized with ethidium bromide, demonstrating specificity.

**Table 1:** Sequences of primers used in Real-time PCR.

Primers	Gene Sequence
mouse CtsK forward	5′-AATACCTCCCTCTCGATCCTACA-3′
mouse CtsK reverse	5′-TGGTTCTTGACTGGAGTAACGTA-3′
mouse MMP-9 forward	5′-CTGGACAGCCAGACACTAAAG-3′
mouse MMP-9 reverse	5′-CTCGCGGCAAGTCTTCAGAG-3′
mouse TRAP forward	5′-CACTCCCACCCTGAGATTTGT-3′
mouse TRAP reverse	5′-CATCGTCTGCACGGTTCTG-3′
mouse NFATc1 forward	5′-GGAGAGTCCGAGAATCGAGAT-3′
mouse NFATc1 reverse	5′-TTGCAGCTAGGAAGTACGTCT-3′
mouse c-Fos forward	5′-CGGGTTTCAACGCCGACTA-3′
mouse c-Fos reverse	5′-TTGGCACTAGAGACGGACAGA-3′
mouse β-actin forward	5′-GGCTGTATTCCCCTCCATCG-3′
mouse β-actin reverse	5′-CCAGTTGGTAACAATGCCATGT-3′

### 4.7. Western Blotting

BMMs were seeded in 6 cm dishes at a concentration of 2 × 10^6^ cells/dish and cultured in α-MEM with 10% FBS and 20 ng/mL M-CSF. Then, the cells were stimulated with RANKL (20 ng/mL) at 37 °C. The cell lysate was prepared using cell lysate buffer (Beyotime, Shanghai, China) supplement with phosphatase inhibitor cocktail and protease inhibitor cocktail according to manufacturer’s instructions. The protein concentration was determined using BCA assay kit (Beyotime, Shanghai, China). An equivalent amount of protein (50 μg) was separated by SDS-PAGE and transferred to a PVDF membrane (Millipore, Billerica, MA, USA). The membrane was blocked with 3% BSA in TBST for 1 h at room temperature. Then, the membrane was incubated with individual primary antibodies overnight at 4 °C. The membrane was washed and incubated with appropriate HRP-conjugated secondary antibodies. The immunoreactive signals were visualized using MicrochemiChemiluminescence system 4.2 (DNR, Jerusalem, Israel).

### 4.8. Statistical Analysis

All data was expressed as means ± SD and analyzed by ANOVA tests using Graphpad Prism 6 software (Graphpad software, La jolla, CA, USA). A *p*-value of <0.05 was considered significant.

## 5. Conclusions

This is the first study to report that calycosin inhibited RANKL-induced osteoclastogenesis from BMMs through down-regulating RANKL-induced expression of c-Fos and NFATc1 in NF-κB and MPAK dependent manners. These results suggested that calycosin has potential as a therapy for diseases associated with bone loss.

## Supplementary Materials

Supplementary materials can be found at http://www.mdpi.com/1422-0067/16/12/26179/s1.

## References

[1] Rho J., Takami M., Choi Y. (2004). Osteoimmunology: Interactions of the immune and skeletal systems. Mol. Cell.

[2] Teitelbaum S.L. (2000). Bone resorption by osteoclasts. Science.

[3] Boyle W.J., Simonet W.S., Lacey D.L. (2003). Osteoclast differentiation and activation. Nature.

[4] Kajiya M., Giro G., Taubman M.A., Han X., Mayer M.P., Kawai T. (2010). Role of periodontal pathogenic bacteriain RANKL-mediated bone destruction in periodontal disease. J. Oral Microbiol..

[5] Akiyama T., Dass C.R., Choong P.F. (2008). Novel therapeutic strategy for osteosarcoma targeting osteoclast differentiation, bone-resorbing activity, and apoptosis pathway. Mol. Cancer Ther..

[6] Teitelbaum S.L., Ross F.P. (2003). Genetic regulation of osteoclast development and function. Nat. Rev. Genet..

[7] Ihn H.J., Lee D., Lee T., Shin H.I., Bae Y.C., Kim S.H., Park E.K. (2015). The 1,2,3-triazole derivative KP-A021 suppresses osteoclast differentiation and function by inhibiting RANKL-mediated MEK-ERK signaling pathway. Exp. Biol. Med..

[8] Takayanagi H. (2007). Osteoclast differentiation and activation. Clin. Calcium.

[9] Takayanagi H., Kim S., Koga T., Nishina H., Isshiki M., Yoshida H., Saiura A., Isobe M., Yokochi T., Inoue J. (2002). Induction and activation of the transcription factor NFATc1 (NFAT2) integrate RANKL signaling in terminal differentiation of osteoclasts. Dev. Cell.

[10] Logar D.B., Komadina R., Prezelj J., Ostanek B., Trost Z., Marc J. (2007). Expression of bone resorption genes in osteoarthritis and in osteoporosis. J. Bone Miner. Metab..

[11] Shen D., Xie X., Zhu Z., Yu X., Liu H., Wang H., Fan H., Wang D., Jiang G., Hong M. (2014). Screening active components from Yu-ping-feng-san for regulating initiative key factors in allergic sensitization. PLoS ONE.

[12] Xu Y., Feng L., Wang S., Zhu Q., Zheng Z., Xiang P., He B., Tang D. (2011). Calycosin protects HUVECs from advanced glycation end products-induced macrophage infiltration. J. Ethnopharmacol..

[13] Chen J., Zhao X., Li X., Wu Y. (2015). Calycosin induces apoptosis by the regulation of ERβ/miR-17 signaling pathway in human colorectal cancer cells. Food Funct..

[14] Zhou Y., Liu Q.H., Liu C.L., Lin L. (2015). Calycosin induces apoptosis in human ovarian cancer SKOV3 cells by activating caspases and Bcl-2 family proteins. Tumour Biol..

[15] Qiu R., Ma G., Zheng C., Qiu X., Li X., Mo J., Li Z., Liu Y., Mo L., Bi G., Ye Y. (2014). Antineoplastic effect of calycosin on osteosarcoma through inducing apoptosis showing *in vitro* and *in vivo* investigations. Exp. Mol. Pathol..

[16] Gong A.G., Li N., Lau K.M., Lee P.S., Yan L., Xu M.L., Lam C.T., Kong A.Y., Lin H.Q., Dong T.T. (2015). Calycosin orchestrates the functions of DangguiBuxue Tang, a Chinese herbal decoction composing of Astragali Radix and Angelica Sinensis Radix: An evaluation by using calycosin-knock out herbal extract. J. Ethnopharmacol..

[17] Ikeda F., Nishimura R., Matsubara T., Tanaka S., Inoue J., Reddy S.V., Hata K., Yamashita K., Hiraga T., Watanabe T. (2004). Critical roles of c-Jun signaling in regulation of NFAT family and RANKL-regulated osteoclast differentiation. J. Clin. Investig..

[18] Matsumoto M., Kogawa M., Wada S., Takayanagi H., Tsujimoto M., Katayama S., Hisatake K., Nogi Y. (2004). Essential role of p38 mitogen-activated protein kinase in cathepsin K gene expression during osteoclastogenesis through association of NFATc1 and PU.1. J. Biol. Chem..

[19] Matsumoto M., Sudo T., Maruyama M., Osada H., Tsujimoto M. (2000). Activation of p38 mitogen-activated protein kinase is crucial in osteoclastogenesis induced by tumor necrosis factor. FEBS Lett..

[20] Mediero A., Perez-Aso M., Cronstein B.N. (2013). Activation of adenosine A(2A) receptor reduces osteoclast formation via PKA- and ERK1/2-mediated suppression of NFkappaB nuclear translocation. Br. J. Pharmacol..

[21] Lee S.H., Kim J.K., Jang H.D. (2014). Genistein inhibits osteoclastic differentiation of RAW 264.7 cells via regulation of ROS production and scavenging. Int. J. Mol. Sci..

[22] Huh J.E., Lee W.I., Kang J.W., Nam D., Choi D.Y., Park D.S., Lee S.H., Lee J.D. (2014). Formononetin attenuates osteoclastogenesis via suppressing the RANKL-induced activation of NF-κB, c-Fos, and nuclear factor of activated T-cells cytoplasmic 1 signaling pathway. J. Nat. Prod..

[23] Luo D., Kang L., Ma Y., Chen H., Kuang H., Huang Q., He M., Peng W. (2014). Effects and mechanisms of 8-prenylnaringenin on osteoblast MC3T3-E1 and osteoclast-like cells RAW264.7. Food Sci. Nutr..

[24] Karieb S., Fox S.W. (2011). Phytoestrogens directly inhibit TNF-α-induced bone resorption in RAW264.7 cells by suppressing c-Fos-induced NFATc1 expression. J. Cell. Biochem..

[25] Shakibaei M., Buhrmann C., Mobasheri A. (2011). Resveratrol-mediated SIRT-1 interactions with p300 modulate receptor activator of NF-κB ligand (RANKL) activation of NF-κB signaling and inhibit osteoclastogenesis in bone-derived cells. J. Biol. Chem..

[26] Wang Z.Q., Ovitt C., Grigoriadis A.E., Mohle-Steinlein U., Ruther U., Wagner E.F. (1992). Bone and haematopoietic defects in mice lacking c-Fos. Nature.

[27] Matsuo K., Galson D.L., Zhao C., Peng L., Laplace C., Wang K.Z., Bachler M.A., Amano H., Aburatani H., Ishikawa H. (2004). Nuclear factor of activated T-cells (NFAT) rescues osteoclastogenesis in precursors lacking c-Fos. J. Biol. Chem..

[28] Kim J.H., Kim N. (2014). Regulation of NFATc1 in Osteoclast Differentiation. J. Bone Metab..

[29] Wada T., Nakashima T., Hiroshi N., Penninger J.M. (2006). RANKL-RANK signaling in osteoclastogenesis and bone disease. Trends Mol. Med..

[30] Bohm C., Hayer S., Kilian A., Zaiss M.M., Finger S., Hess A., Engelke K., Kollias G., Kronke G., Zwerina J. (2009). The α-isoform of p38 MAPK specifically regulates arthritic bone loss. J. Immunol..

[31] Nakamura H., Hirata A., Tsuji T., Yamamoto T. (2003). Role of osteoclast extracellular signal-regulated kinase (ERK) in cell survival and maintenance of cell polarity. J. Bone Miner. Res..

[32] Kim J.Y., Cheon Y.H., Kwak S.C., Baek J.M., Kim Y.C., Yoon K.H., Oh J., Lee M.S. (2014). 9-Hydroxy-6,7-dimethoxydalbergiquinol inhibits osteoclast differentiation through down-regulation of Akt, c-Fos and NFATc1. Int. Immunopharmacol..

[33] Jimi E., Aoki K., Saito H., D’Acquisto F., May M.J., Nakamura I., Sudo T., Kojima T., Okamoto F., Fukushima H. (2004). Selective inhibition of NF-κB blocks osteoclastogenesis and prevents inflammatory bone destruction *in vivo*. Nat. Med..

[34] Wang Y., Xu X., Wang H.B., Wu D., Li X.O., Peng Q., Liu N., Sun W.C. (2015). 17-Hydroxy-jolkinolide A inhibits osteoclast differentiation through suppressing the activation of NF-κB and MAPKs. Int. Immunopharmacol..

