# Supplementary material for: Calycosin Suppresses RANKL-Mediated Osteoclastogenesis through Inhibition of MAPKs and NF-κB
Source: Int J Mol Sci. 2015 Dec 10;16(12):29496–507. doi: 10.3390/ijms161226179 (PMC4691122; doi:10.3390/ijms161226179)
Supplement: Supplementary file 1 [file ijms-16-26179-s001.pdf]

Supplementary Material: Calycosin Suppresses RANKL-Mediated Osteoclastogenesis through Inhibition of MAPKs and NF-κB

Gui-Hua Quan, Hongbing Wang, Jinjin Cao, Yuxin Zhang, Donglin Wu, Qisheng Peng, Ning Liu and Wan-Chun Sun

NMR Spectrum and MS Data for Calycosin

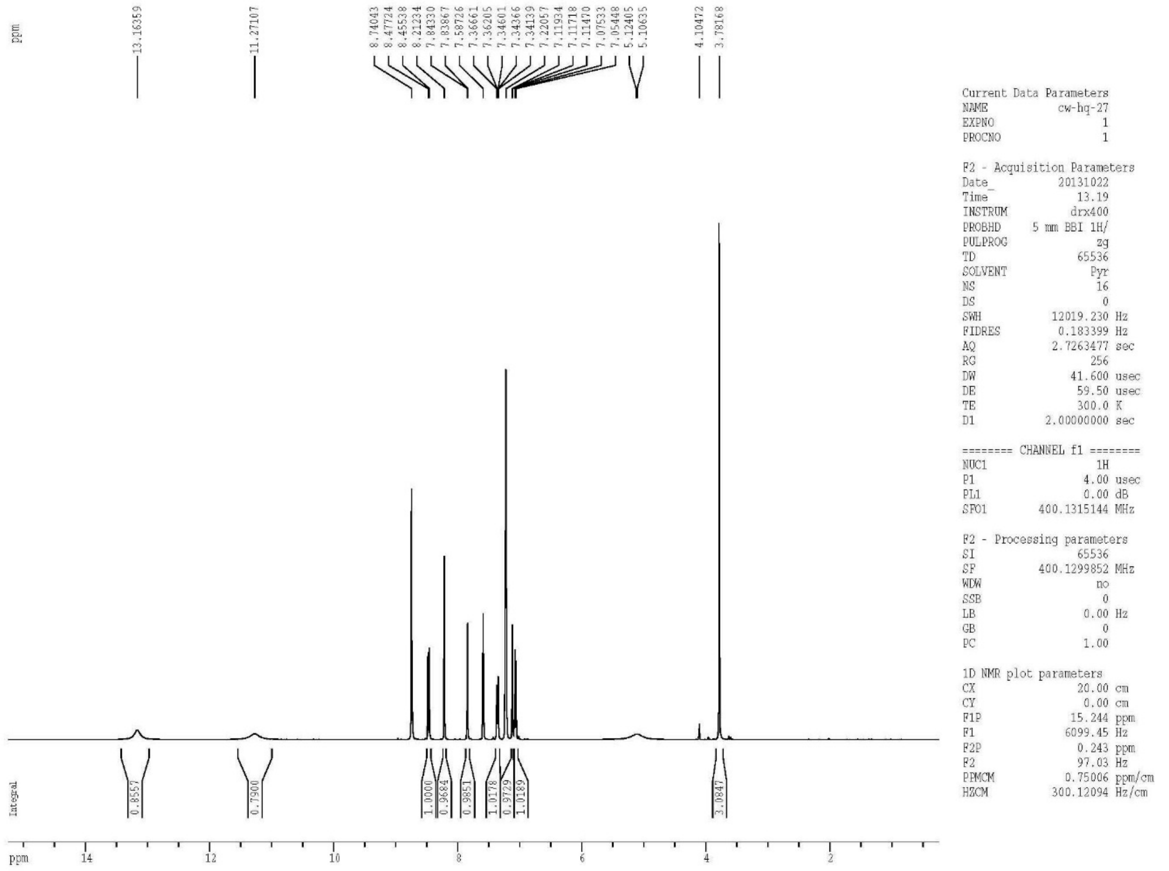

Figure S1. ^{1}H-NMR (400 MHz, pyridine-d_5) spectrum of calycosin.

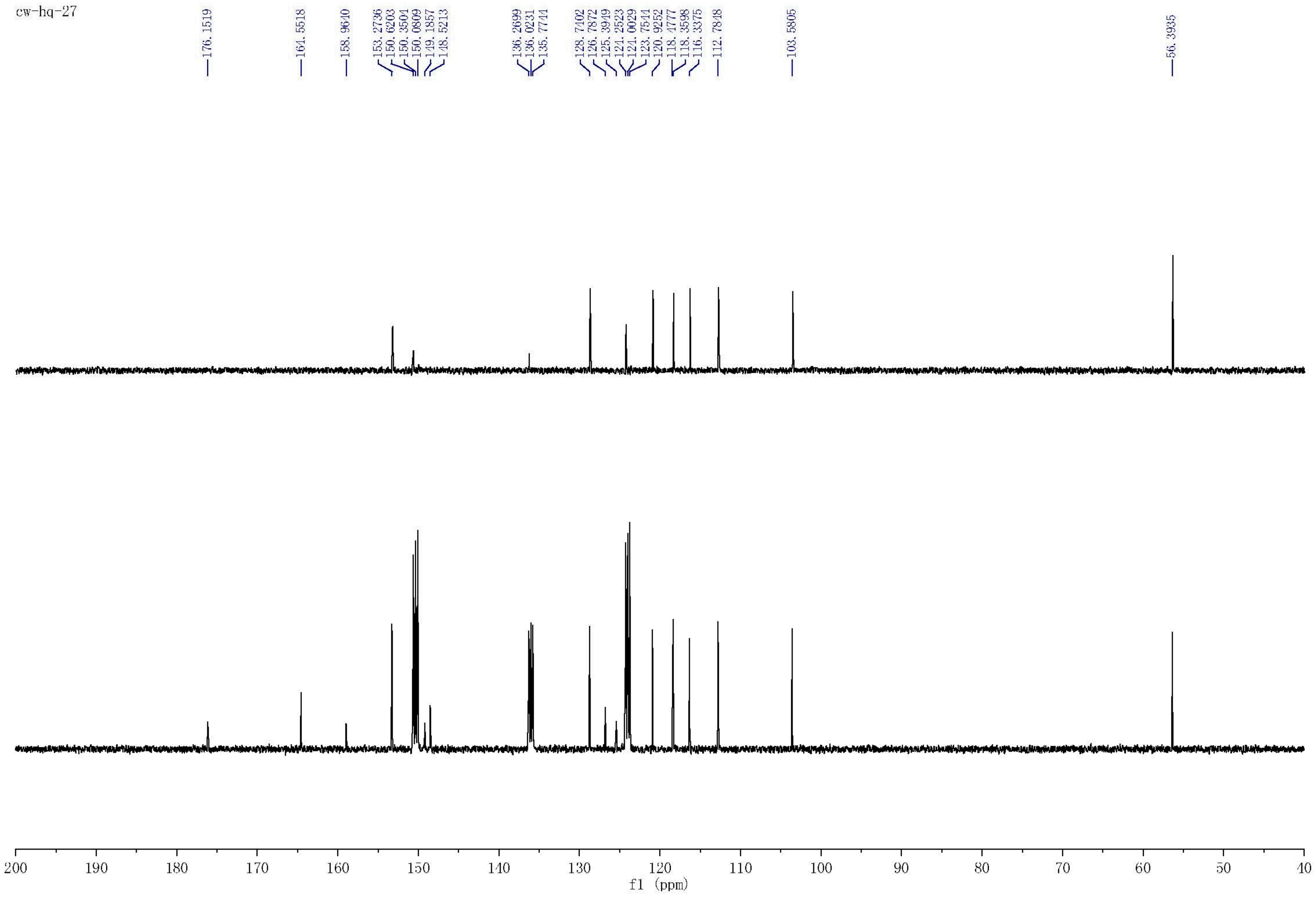

Figure S2. ^{13}C-NMR (100 MHz, pyridine-d_5) spectrum of calycosin.

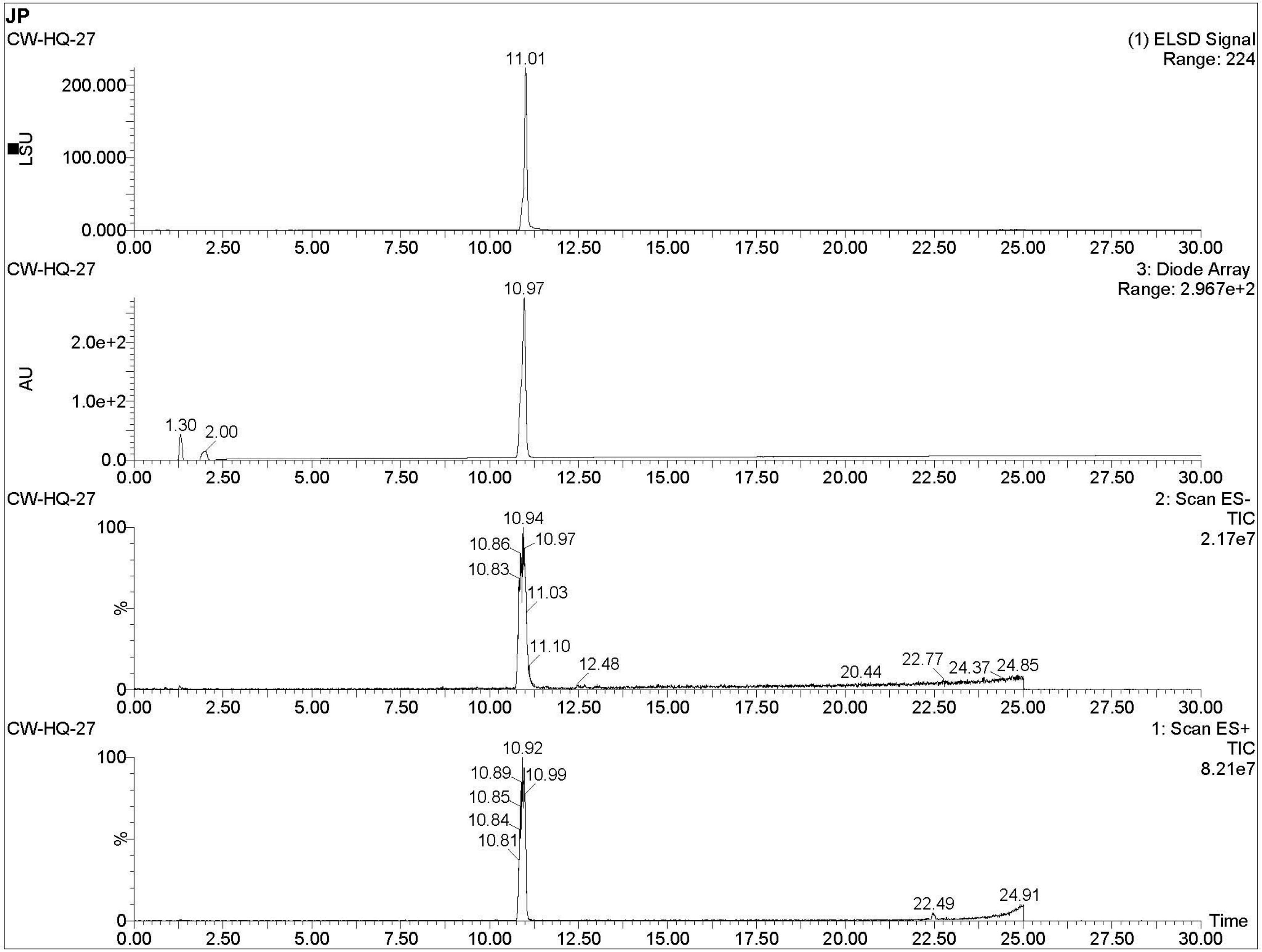

Figure S3. *Cont.*

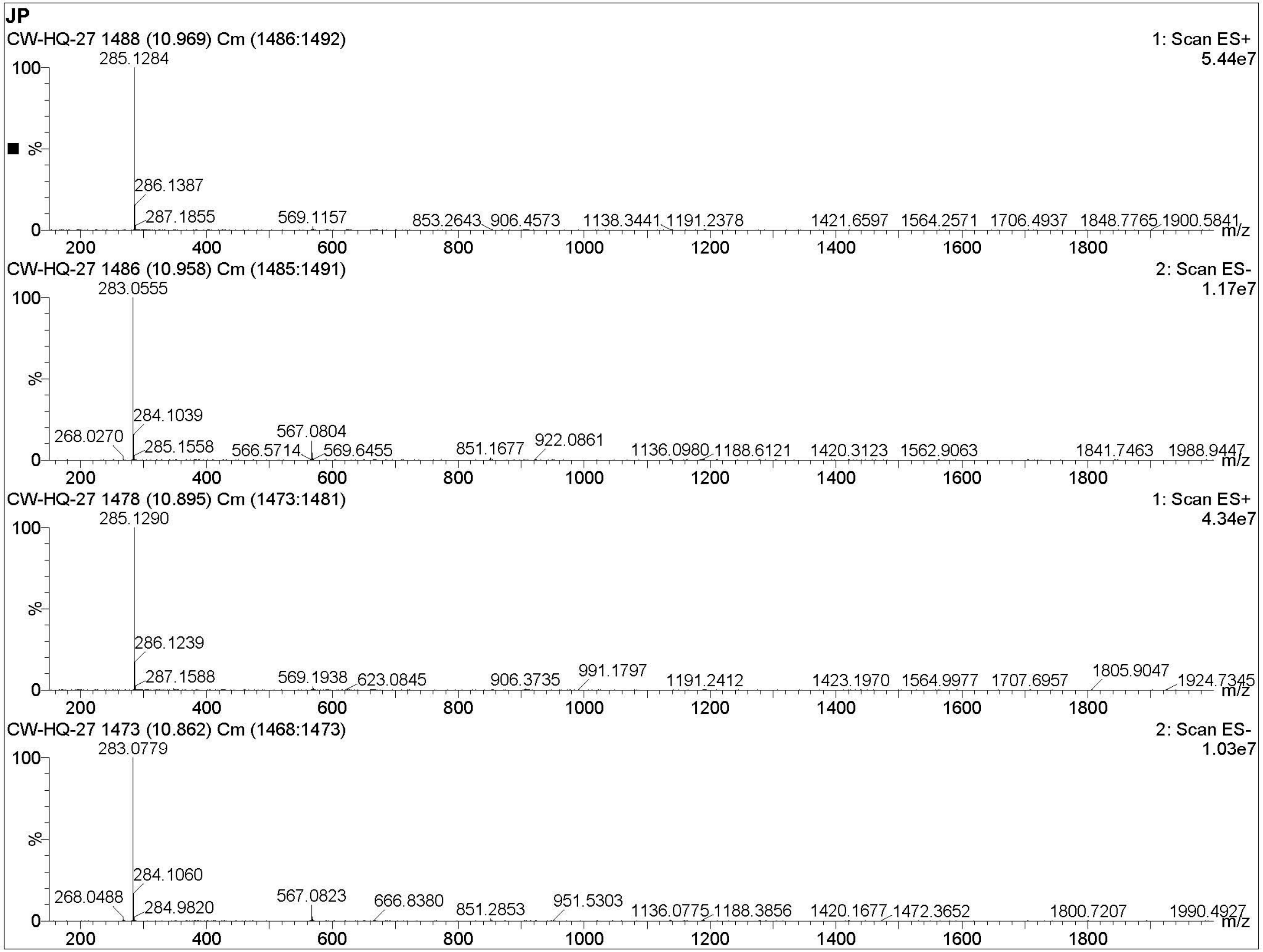

Figure S3. LC-MS spectrum of calycosin.
